# The Hospital Nursing Supplement

**Published:** 1893-04-15

**Authors:** 


"The Hospital" Nursing Mirror.

Being the Extra Nursing Supplement of "The Hospital" Newspaper.

[Contributions for this Supplement should be addressed to the Editor, The Hospital, 428, Strand, London, W.C., and should have the word "Nursing" plainly written in left-hand top corner of the envelope.]

### News from the Nursing World.

### THE PRIVY COUNCIL AND THE CHARTER.

We understand that the decision of the Privy Council, with regard to the charter applied for by the Royal British Nurses' Association, will probably be made public after the first Council meeting following the Queen's return from the Continent.

### CHARITY AND CHEERFULNESS.

Their Royal Highnesses Prince and Princess Christian are announced as the patrons of the private subscription ball to be given at the Whitehall Rooms on the 19th inst. The object of the entertainment is the raising of funds for the Charterhouse Mission, and for Princess Christian's own Trained Nurses' Home at Windsor.

### ROYAL NATIONAL PENSION FUND FOR NURSES.

An interesting fact is brought out in connection with this Fund by the tables contained in the Life Insurance Blue Book just issued by the Board of Trade. The proportion of expenses and commissions to premium income, on the whole of the 101 ordinary and industrial companies included in the tables for the year 1892, was 22·8 per cent. Only eight of these 101 companies expended less than 10 per cent. of the premium income upon expenses, of which the Royal National Pension Fund for Nurses was one. The proportion of expenses to premium income in the case of this Fund has fallen from 10·6 per cent. in 1891 to 7·7 per cent. in 1892. These figures are very satisfactory, as they show the great economy practised by the Council. They should increase the confidence of the nurses in the Pension Fund in which Her Royal Highness the Princess of Wales takes so deep and active an interest.

### REST AND RECREATION.

People talk about "nurses' holidays," and what they ought to consist in, as if all trained women liked to rest after exactly the same fashion. *The Trained Nurse* has a good letter on the subject this month, in which the writer remarks: "A nurse's recreation should be suited to her capacity for enjoyment. Taking into consideration the many and diverse tastes of womankind . . . it will be readily seen that the recreations must be many and varied." Holidays should not only be restful, they should benefit the mind as well as the body. Fresh interests should be taken up, and it does not matter much what these are, so long as they replace the ordinary daily occupations, and are entirely different ones. Absolute change of place and employment constitutes the best kind of holiday for busy workers, sending them back mentally and physically braced to renew the incessant conflict against disease and suffering. Moreover, a wisely spent vacation is found to be the best safeguard against that narrow-mindedness which is the trained nurse's greatest snare.

### NURSING AT PLAISTOW.

In the record of parish work which the Rev. T. Given-Wilson has recently set forth in an interesting little pamphlet, nurses have a prominent place. St. Mary's, Plaistow, is a huge parish of 21,000 people, "of whom certainly but very few could by any stretch of imagination be called well-to-do." "There were two developments of the work in Plaistow which did more than anything to humanise the place, and to win the hearts of the people—nursing was one." And the nursing in St. Mary's parish is as thoroughly well organised as every other department there. All the nurses are trained and certificated, and there are now thirty at the home, which was only opened in 1891. They visit the sick by night and by day in their own homes, and admit the worst cases to the Cottage Hospital. "They have soothed many a sufferer, and have made the departure for that journey through the Dark Valley as peaceful as poor humanity can make it." The nursing at Plaistow, organised by Miss Katherine Twining, has become so popular that people even come and settle in the parish for the avowed purpose of sharing in its advantages. The little hospital has a resident medical officer.

### HIDDEN DANGERS.

Amongst many fruitful sources of infection unprofessional persons would hardly think of enumerating "patients' friends." Hospital nurses, however, give this cause its due importance, and not infrequently trace the importation of an epidemic into a sick ward to the imprudence or ignorance of one visitor. The so-called "sterner sex" does not realise this danger as clearly as do thoughtful nurses. The former seize on the sentimental view of the subject, and prefer to risk the infection of people, and especially children, already ill, rather than encounter the unpopularity which might possibly be incurred by a stricter limitation of "visitors' days." In England patients with typhoid have the same number of visitors as those suffering from less serious diseases, and all nurses know the anxious watchfulness needed to restrain the kindly relatives from surreptitiously administering such "relishes" as would soon place the unlucky patient beyond the reach or need for "skilled nursing." The authorities at Prince Alfred Hospital, Sydney, with wiser policy, restrict the friends of typhoid patients to one visit a week, thereby greatly increasing the sufferer's chances of recovery,

### CAMBRIDGE.

The Committee of Addenbrooke's Hospital are aware of their own good fortune in having a Matron who is as competent as she is popular, and they unanimously agreed at a recent meeting to raise her well-earned salary. By doing this they hope to retain her valuable services for the hospital, which is now undergoing such extensive structural improvements.

### LADIES TO THE RESCUE.

The people of Bradford doubtless do much good work, but they will have to take some vigorous measures to secure success to their district nursing. On January 14th, under the title, "Half-hearted Benevolence," The Hospital called attention to the strange arrangement which permitted the fair gains of the private nurses to be absorbed in defraying the cost of another contingent of workers. At the annual meeting of subscribers to the institution, held on the 28th ult., the Mayor urged the need for some change, and the Rev. J. S. Addison expressed his regret at charitable work being defrayed to a large extent out of the nurses' earnings, and of the importance of removing "such a stigma from the institution." Drs. Dunlop and Mossop spoke warmly of the district nurses' work, and heartily concurred in Mr. Addison's opinion. It is to be hoped that the proposed Ladies' Committee will be able to reorganise matters, and to raise funds for charitable purposes in a less questionable fashion.

### PRACTICAL SANITATION.

Under the auspices of the Belfast Health Society Miss J. M. S. Tod gave an excellent address last month. Her sound remarks on the obligations of all women to acquire practical knowledge of the construction of their own dwellings were most helpful to her intelligent hearers. *The Belfast Health Journal* gives a summary of her speech, calling attention to the fact that the "supply and preparation of food, the repair and renewal of clothing, the keeping of rooms and furniture in order, the care of children, of the aged and infirm, and the nursing of the sick," are duties laid upon every ordinary mother. Under such circumstances, the more practical the preliminary training which each woman secures for herself, the greater the benefit, not only to her household, but eventually to the whole community. "Many a woman," said Miss Tod, "both in large houses and small, is made anxious by recurring evidence of the presence of bad air, yet she is ignorant that the conditions required for health are also the conditions of comfort." What is wanted, therefore, is systematic instruction in hygiene and sanitation for all classes of women. This would also largely aid in the banishment of preventable diseases.

### SUCCESSFUL CANVASSING.

Many advertisements inform intending candidates for the office of matron that any personal canvassing will disqualify them. This warning appeals to our sense of justice, even though it disappoints ladies who have confidence in their own personal influence. Evidently no such stipulation was attached to the vacancy at the Clare County Infirmary, and the free discussion which took place at the special meeting of the Committee of Management reveals a somewhat curious administration. Three or four applicants for the matronship were very well recommended, but the interest of the meeting centred round Miss Sigerson. This lady called previously on several members of the committee, including the chairman, and whilst one gentleman spoke of being "most favourably" impressed by her, another went farther, saying he found her "most fascinating," and "spent a very agreeable half-hour with her." Miss Sigerson, however, had another attraction; she set a low price on her services. She gave "an undertaking" to Mr. F. F. Cullinan "to accept the office, if selected, at £40 per annum." The salary offered being £50, the ratepayers would save £10 by the selection of this fascinating candidate. After some discussion, and a little opposition, Miss Sigerson was chosen, the chairman giving the casting vote. Reforms of various sorts are needed in Clare Infirmary, for the insubordination of the domestic staff is somewhat notorious, the last matron being lately informed by the servants "that they were not subject to her at all, that they had not been appointed by her." Unless Miss Sigerson's position is more justly defined she will hardly find it a comfortable one.

### REDUCED ACCOMMODATION.

It is feared that the depressed state of the finances of the Women's Hospital at Melbourne will necessitate the closing of some of the wards. Such a proceeding would be disastrous, for over a hundred patients are waiting for admission, all cases requiring operations, and some of them so urgent, that it is doubtful whether they can live until beds are vacant for them. With such pressure as this on the existing accommodation, it is indeed sad to think that any reduction in the number of beds should have to be contemplated.

### A NURSES' HOME FOR ORANGE, U.S.A.

The inhabitants of Orange, U.S.A., have awakened to the fact that a Home for Nurses is sorely needed in their city. There are now sixty-three graduates working in Orange, and they have no settled head-quarters, many being scattered in boarding-houses, and difficult to find when required. The total cost of the undertaking is estimated at 12,500 dols., and towards this 1,055 dols. is already secured. Doubtless the whole sum will be forthcoming ere long, and the house which it is under contemplation to purchase, will become a Nurses' Home, in the best sense of the word.

### SHORT ITEMS.

The east wing of the Perth Royal Infirmary contains two large wards which serve for cases of measles. One of them has been now arranged for the reception of cholera patients, who will be nursed there if the disease appears in the district.—After praising the self-sacrificing work of the doctors at Rainhill Asylum the other day, Mr. Snape, M.P., referred to the possibility of Parliament being asked to authorise the County Councils to pay for the training of nurses. Presumably he meant for the County Asylums.—Miss Fenwick Bisset has provided a year's salary for an additional nurse for Aberdeen, who will enter on her duties this month.—It has been decided to make some alterations and additions to the Home for the Diocesan Trained Nurses at Salisbury, at a cost of £700; a bath-room is one of the proposed improvements, and we are sorry to think that any home accommodating thirty nurses should have been so long without this necessity.—The Nurses' Syndicate, founded a short time ago in Paris by Madame Constant, already numbers 600 members.—The Workhouse Infirmary Nursing Association reports that 33 Boards of Guardians accepted the system of trained nursing, up to the end of last year, and 20 more applications have been received from new Boards.—Dispensing as well as midwifery and monthly nursing can be learnt in connection with the Clapham Maternity Hospital, Jeffries Road.

### THE DISABLED NURSE.

We have to acknowledge 2s. from "S. W.," making total £12 10s. Of this £4 17s. 6d. has been handed over to the Disabled Nurse.

### THE HOSPITAL ENDOWED BED.

We have received this week 30s. from "L. W.," £1 from Mrs. Bullock, and 10s. 6d. from Miss Heanley, making £17 18s. 6d. up to present date.

### Sanitation for Nurses.

By a Sanitary Inspector.

### I.—INFECTIOUS DISEASES.

The duties and responsibilities of a nurse engaged in district work are so various, and so different to those of one employed in a hospital, that we propose to give some hints on one particular form of knowledge which is needed by the district nurse, but which her sister in a hospital is not called upon to have. This refers to the subject of the laws which relate to infectious diseases.

It may often happen that a nurse is attending a patient in her district, perhaps some case of chronic disease, or some bed-ridden old man or woman, whom the doctor has gladly given over almost entirely to her care, and whom he does not often visit. One day, on paying her usual call, she finds one of the children, or perhaps an adult, in the same house, ill with what appears suspiciously like an infectious disease, and the question arises, What does the law require to be done under such circumstances?

At the beginning of last year a new Act of Parliament, known as, the Public Health (London) Act, of 1891 came into force, containing more stringent regulations as regards infectious diseases, as well as many other matters, than were to be found in its predecessor, namely the Public Health Act, passed in 1875. This new Act makes compulsory much that before, was either optional, or allowed a good deal of freedom, and it is to be hoped that it will soon prove of great use in lessening the spread of infectious diseases—a danger which at present is largely due to carelessness arising from absolute ignorance amongst the poorer classes, especially of what their duty to their neighbour is, when infectious disease breaks out in their houses. In the spreading of this most useful knowledge the district nurse will be invaluable if she will but take the necessary trouble to acquaint herself with the regulations which have so carefully been laid down for the guidance of those concerned.

As soon as there is suspicion of the presence of an infectious disease, if a doctor is not already in attendance, it is the duty of the head of the family, or of the nearest relative present in the house at the time, to give notice to the medical officer of health of the district in which the patient happens to be.

Failing the nearest relative, then the duty devolves on any person in charge of, or in attendance on, the patient; but if there should be no such person, then the master of the house must do so.

The nurse's actual responsibility does not go beyond either seeing that such notice be given by the proper person, or having herself to perform that duty. Neglect to notify an infectious disease incurs a fine of forty shillings. The sanitary authority of the district supplies regular certificates for the doctor in attendance to fill in, and he is required to certify the disease from which he believes the patient to be suffering, together with the full name and address, the sex, and age of the patient. Such certificate can, of course, only be filled in by a fully-qualified medical practitioner.

It may be well here to explain what is meant by the term "Sanitary Authority."

According to the definition in the Act, it refers to different municipal bodies, acting in the particular district of London, but it includes the Commissioners of Sewers in the City of London, the vestry of the parish, or the district board for any particular district, or the Local Board of Health, or the Board of Guardians, or the Overseers of the poor. All or any of these have to see that the Act is properly carried out in their district. At any rate, in the case of infectious diseases, it is the medical officer of health with whom private individuals have to communicate.

The Act specifies certain infectious diseases which it declares to be "dangerous," and which have to be duly notified.

These are small-pox, scarlet fever or scarlatina, erysipelas, typhoid or enteric fever, typhus, continued, relapsing, puerperal fever, diphtheria, cholera, and membraneous croup.

The Sanitary Authority, however, can, if deemed advisable, call a meeting of its members and pass a resolution that any other disease they choose to specify shall be notified in the district over which they have authority, and such a resolution, after being approved by the Local Government Board, and duly advertised in the local papers, and generally made known in the district by handbills during the space of one week, shall come into force in that particular district only, either temporarily or permanently. If the order is only to be temporary, this is to be distinctly stated, and also the exact period during which it is to be enforced.

By this means both measles and influenza have been made notifiable in certain districts, and all district nurses should be aware what diseases are to be notified.

In the case of a child who is going to a school, having one of the infectious diseases specified above, or of children who are attending school being in the same house as the patient, it is now required by the new Act that notice be sent also to the head teacher of the school attended by the children, which ought to do much to prevent the spread of infectious disease.

We, ourselves, should be very glad to see measles included amongst the "dangerous" diseases which must be notified, for certainly amongst the poor this disease makes sad havoc, and is decidedly dangerous in the sense of causing a high death rate. Amongst the rich and well-to-do measles is not considered a very grave trouble, unless the child happens to be a delicate one and unable to resist disease.

Amongst the poor it is quite otherwise, for even temperature, freedom from draughts, sufficient nourishing food and good clothing cannot be insured, much less a proper supply of pure air and space. Of course, notification without the provision of sufficient accommodation in a hospital for infectious diseases, is quite absurd, and merely supplies accurate statistics for those who delight in such things, but it does nothing towards stamping out disease. The same sanitary laws are in force in Scotland as in this country, though the Scotch show their characteristic thoroughness and common sense in the care and minuteness with which they carry out all details connected with the prevention of the spread of infectious diseases, with the satisfactory result that the percentage of mortality in Scotch towns compares very favourably with that of others in England.

In Edinburgh especially, notification of infectious diseases is carried out very thoroughly, and we are glad to find that measles are notified, and the inspector "calls at once at the house, sees to means of isolation, warns parents about keeping children at home, tells them what precautions to take, and to send to him when the house is ready for disinfection, if it is needed." All this reads very well, but what is the use of telling poor people what to do without giving them the means of carrying out directions? Suppose a family of five or six children, some of the elder ones being able to work, and the father also, and all living in three or four rooms. If one of the younger children gets measles, in all probability the other children who are at work will have to stay away—certainly those going to school will have to remain at home; and it is almost certain the whole family will be laid up with measles, as under the circumstances the first child could not possibly be isolated at home. No doubt the proper isolation of measles will put a heavy strain on hospital accommodation at first, though we may fairly hope that when the measure has been in force for a time, the disease will become less common than at present. At all events, it certainly appears that such an anomaly as notification without isolation ought to be promptly abolished.

*(To be continued.)*

### Test Questions

GIVEN TO NURSES OF THE STATE HOSPITAL, UTICA, NEW YORK, PRIOR TO THEIR EXAMINATION—(*continued*).

Last week we presented to our readers the first half of the Test Questions which were given to the nurses at the State Hospital, Utica, and with the subjoined list they certainly form a rather imposing array of queries. However, it must be remembered that these are not meant as examination questions in the common use of the term, but rather in preparation for them. They must be of immense practical help to students in showing them wherein their own weakness lies, as well as in pointing out the kinds of subjects requiring study. There is a commendable thoroughness in the system, although, of course, some questions are more useful to nurses than others. Still they all go to encourage thoughtfulness and a desire for increased knowledge, two very desirable ends to attain.

Escape, Sunstroke, and Frost-bite.

41. Why is it advisable not to care for an insane man at his home?

42. Mention some of the dangers of escape to the patient himself.

43. How and why should you learn the peculiarities of a patient coming to your ward?

44. What is it best to do in taking patients to and from a ward?

45. Why should you be careful not to allow a patient to even handle a key?

46. What is it advisable to do in regard to learning the names of patients, and why?

47. In a general way, what is to be done when a patient escapes?

48. What are you to do when you have found him, and what have you to guard against?

49. Tell what you can of the two kinds of sun-stroke, the bodily condition, prevention, cause, and treatment.

50. The same about frost-bite.

Fire, Burns, and Scalds.

51. How do you operate a fire extinguisher?

52. What is the reason for so much care of the fire pails?

53. What three things are you to think of when a fire is discovered?

54. What do you have nearest at hand in case of a fire in the wards?

55. What would be your method in taking patients from a burning ward?

56. What from other wards?

57. What do you have to guard against in the matter of using stairways at the time of the fire?

58. What should we be absolutely sure of before finally leaving a burning ward?

59. Tell how you would treat a severe burn or scald.

60. Tell what you can about choking, its prevention, causes, and treatment.

Suicide and Artificial Respiration.

61. In what two ways may a person be predisposed to suicide?

62. Give examples of suicidal impulse?

63. Among what class of patients do most suicides occur?

64. How does a melancholy patient think out his suicidal determination?

65. What do we have to think of besides recovery when a melancholy patient suddenly becomes cheerful?

66. What do you do to prevent suicide?

67. How would you perform artificial respiration after drowning and after strangulation, and how would you be guided in your work, and what dangers would you avoid?

68. What would you do a in case of cut throat or cut arteries?

69. How would you treat a patient whom you thought had taken poison?

70. What is it that we should have always in mind, and strive always to carry into effect, regarding emergencies?

General Nursing.—First Lecture.

71. What should be noticed about pain?

72. What should be noticed about the skin?

73. When does the blood receive oxygen?

74. What is the object of ventilation?

75. What are the best means of ventilating an ordinary room?

76. At what temperature should the room be kept for fever patients?

77. How would you dust a room?

78. How would you make up a bed?

79. How would you prevent bedsores forming?

80. What are the first symptoms of bedsores?

General Nursing.—Second Lecture.

81. What is the normal temperature of the body?

82. How is the temperature of the body taken?

83. What temperature indicates high fever?

84. How do you count the pulse?

85. What is the rate of respiration in health?

86. How would you count the respirations?

### Australia.

PRINCE ALFRED HOSPITAL, SYDNEY.

Lectures on invalid cookery form an excellent feature in the training of probationers at this fine hospital. The course last year was given by Miss Keegan. Lectures to the nursing staff on Elementary Anatomy and Physiology, Medical and Surgical Nursing, Ophthalmic Cases, Children, Materia Medica, Nursing of Insane, Delirious and Nervous Patients, and Gynæcological Cases; also the application of Electricity and Massage, were held by Drs. Jenkins, MacAllister, and Scott-Skirving, Professor Wilson, Drs. Thring, Graham, and Purser. Many applications to join the nursing staff are received, and well educated young women are selected to fill vacancies. The new Nurses' Home, opened by the Countess of Jersey on December 13th, is occupied and appreciated, the arrangements being particularly comfortable and well managed. The exceptional abilities of the Matron, Miss McGahey, are universally acknowledged, and her power of successful organisation has had full scope. As the report says: "The Matron has devoted herself with much energy, self-sacrifice, and ability to the numerous duties of her department, to the development of efficiency in the nursing staff, to advance the well-being of the nurses, and to increase their material and social comforts. She has also taken great trouble and interest in the arduous task of organising the Nurses' Home." We wish continued success to the managers of this beautiful hospital, and trust the present financial depression will not necessitate the closing of any wards in an institution where all the beds are urgently in demand.

### Minor Appointments.

Monsall Fever Hospital.—Miss Mary Elliot has been made Night Superintendent at Monsall Fever Hospital, near Manchester.

### Everybody's Opinion.

### WOMEN INSPECTORS.

NURSE JANETTA writes: I am very much interested to see in the papers that the County Council have made a woman an "inspector" at Brighton. I believe she is to visit houses where the children have been ill. I should be glad to know if any trained nurse has an equal chance of getting a similar post? Also whether Nurse Ramsden has any certificate for proficiency in sanitary knowledge. It seems such a capital field of usefulness for competent nurses, that information on the subject would be welcomed by many readers of THE HOSPITAL.

### "ATTAINABLE PERFECTION."

"S." WRITES: I have read your correspondent's remarks on private nurses with great interest. After an experience of nearly eighteen years in that capacity, I claim to have some knowledge of what a private nurse's life really is. It differs totally from hospital work, and decidedly requires special training. Many people do not look upon the duties of a private nurse as being noble or self-sacrificing, and I remember the late Mrs. Wardroper remarking on one occasion, "There is no honour or glory in nursing a rich person." "Yet," as I said to her, "I often find a rich person in a deplorable state, needing sympathy and care from a skilled nurse quite as much as a poor person." Mrs. Wardroper could not see that. As regards my own experience, I have long seen that if I wish to live peaceably I must try to make the best of existing circumstances, and must not think I am going to set the whole family right in every house I go into, and make them conform to my views. On the contrary, I have often to await their pleasure and humour their little whims and peculiarities. I invariably find by yielding to my patients' wishes they are quite ready to listen to any suggestions I have to make. It often gives me great pleasure to find how much confidence they have in my advice.

### Hints for Nurses.

THE simple little apparatus of which we spoke under the heading of "Ingenious Disinfection" in THE HOSPITAL of April 1st can now be obtained from Messrs. Boake Roberts and Co., Stratford, London. This convenient method for the diffusion of sulphurous acid gas will certainly commend itself to all nurses who find themselves personally responsible for complete disinfection of rooms or clothing.

### Notes and Queries.

### Queries.

(104) *Certificate.*—How can I get a certificate from Medico-Psychological Association?—*Mental Nurse.*

(105) *Training.*—Where can a probationer get training under 23 years of age?—*A. M. L.*

(106) *Disinfection.*—Please say where the apparatus which Dr. Wynter Blyth recommends for this purpose can be obtained.—*Private Nurse.*

(107) *Asylum Attendants.*—Book for instruction wanted.—*Nurse M.*

(108) *Stewardess.*—Where can I get information?—*Nurse Edith.*

### Answers.

(104) *Certificate (Mental Nurse).*—Write for all particulars to Secretary of the Association.

(105) *Training (A. M. L.).*—Get "How to Become a Nurse," by Honnor Morten, Scientific Press, 428, Strand, price 2s. 6d.

(106) *Disinfection (Private Nurse).*—From Messrs. Boake, Roberts, and Co., Stratford, London.

(107) *Asylum Attendants (Nurse M.).*—"Mental Nursing," by Dr. Harding, Scientific Press, 428, Strand, price 2s. 6d.

(108) *Stewardess (Nurse Edith).*—Write to shipping companies and ask.

### Wants and Workers.

NURSE C. would be much obliged for information of a "House of Rest" for clergy, or of any place in the country where a convalescent clergyman could be taken.

NURSE M will feel grateful to anyone who can give a letter for the Provident Surgical Aid Society to a respectable young woman who needs an artificial foot, so that she may be enabled to take a suitable situation.

### For Reading to the Sick.

### SPRING TIME.

WE are all inclined to think how good life would be without disagreeables; but if there were no sickness, no misfortunes, no ill-tempers of any kind; if the sun always shone, and the wind always blew gently from the south, the world would not be so happy as it now is. Contrast makes beauty, and we should tire of the monotony, whilst we should undervalue health and other blessings when there was no fear of losing them. For winter is necessary—nature must rest; she must withdraw her forces for a time to recruit her strength. The nipping frosts, the rude winds which break off the useless branches prepare the way for the sweet pleasures of spring so exquisitely described in the Song of Solomon—"The winter is past, the rain is over and gone; the flowers appear on the earth; the time of the singing of birds is come, and the voice of the turtle is heard in our land; the fig tree putteth forth her green figs, and the vines with the tender grape give a good smell."

Nature is the same in all her actions, and so the human soul requires its winter to prepare it for the pleasures which our Heavenly Father has in store for it. Perhaps some of us are even now going through the winter of our lives; wasting sickness has taken away our vigour, and the relish which we erewhile possessed for all we did is gone. Our hearts, once so full of peace, and love, and hope, that they sang for very joy, are now sad and still from disappointments which have passed over them like a withering blight. Dreary and dull and hopeless we may be, but the season will not last; it will surely pass away, yet not, let us hope, till it has taught us many helpful lessons. But it is useless to try to hurry winter. However impatient we are we cannot shorten its stay by a single hour; we do no good by fretting or chafing or repining. It may seem very hard to wait patiently till our winter is over and gone and our spring time come again, but we must try to acquire the blessed habit of patience, and beguile the sameness of our lives by looking out for the beauties of the present, which even winter brings. And they are easy to find. The crisp air, the glorious star-light nights, the lovely forms made by the hoar frost on tree and window-pane, the cosy, comfortable fires round which our families gather, these are alleviations which come into the saddest lives. Many lives which appear to outsiders to be the most dark and cheerless, are really the opposite, to those who by God's help have determined to make the best of them. It is wonderful how many pleasures we find if we have set our minds on seeking for them. On the other hand, we should also ask ourselves whether there is anything in ourselves to cause our trials? Do we love God? Do we love Christ? Perhaps it is this want of love which is the bane of our lives. Jesus would have our warm personal love; for this He longs, as the hart desireth the water brooks. To win it He came on earth, for it He bore our sins and died our death. Alas! we are cold and do not give it Him. Seeing this, our Father sends us sorrow and suffering because it is the only way by which we can be driven into His bosom. And when we have responded to His love, He will call us to come away, for there are better things to be had with Him, than ever eye beheld, or ear heard, or the heart of man conceived, and when we rise up and follow Him we shall find, "Lo, the winter is past, the rain is over and gone; the flowers have appeared on the earth, and the time of the singing of birds is come."

### Presentation.

ON leaving the Bristol General Hospital Miss Bann, who had held the office of Matron for over eight years, received some very handsome gifts from the medical staff, House Committee, nurses, officers, and servants. A massive gold brooch with B. G. H., set in diamonds, a gilt bronze Empire clock, and an afternoon tea service, with kettle and tray, and a lady's travelling bag were presented with a kindly ad dress which largely added to the value of the gifts. Liverpool is to be congratulated on securing a lady whose record of previous work gives good promise for her future.

### Our Examination Questions.

The question for April, "What are the special points to be observed in the Making and Applying Poultices and Fomentations, and how often should either be changed when no special order is given?" has proved a popular and successful one. We have received a large number of answers, and some thirty or forty of these we can praise.

They certainly prove the writers to have an intelligent knowledge of the end to be aimed at in the application of poultices and fomentations, and most of them also show in a very pleasant manner that the comfort of the patient is the first consideration, the nurse having no wish to spare herself any trouble in securing this.

We are particularly glad to find many infirmary nurses amongst the competitors, and this clearly proves the progress which is being surely though slowly made in the nursing departments of workhouses. Private and district nurses also wisely avail themselves of an opportunity for exhibiting theoretical knowledge and practical skill; indeed papers have reached us from many far away places.

One correspondent, whose answers are not altogether correct, yet develops a special line of her own, for she gives information as to which vessels are best to boil water in when special haste is required, and other useful little details, and we fancy she will make a capital nurse when she has had complete training.

A great uniformity of opinion exists as to the time poultices should remain on, most writers say four hours for large and two for small ones. A few answers give shorter periods, and certainly in acute illnesses it is well to change them much more frequently.

Even good poultice makers do not always realize that the application should be changed whilst it is still warm. If allowed to remain until cool it is probably doing harm. It is so common for patients of all classes to say, "Oh, I'm sure it is not cold yet," as if that were a desirable condition of things to wish for.

On the other hand, a person who is very ill will often find the poultice soon grows colder than his own feverish skin, but will not think of complaining of the discomfort unless the nurse has previously invited him to do so.

One writer considers that a poultice should not be changed when the patient is disinclined for it, but that is probably an uncertain rule to go by. Sick people are frequently unwilling to be disturbed even when it is urgently needful for them to receive attention.

The "leaving alone" policy would therefore be unwise and unsafe, because even the discomfort of a cold poultice would hardly incline a very weak patient to willing concurrence in its removal. Everyone should study to make the changing of poultices as free from fatigue as possible, and this object can be attained by practice and care. A finished nurse should aim at substituting a new for an old poultice without disturbing and even without waking a patient. Difficult as this may sound, it not only has been, but in many cases can be easily done with patience and tact.

The domette and flannel bandages which most of our correspondents advise for keeping poultices in place, are by no means the best metheds to use. The removal is as fatiguing as the application of these yards of material, and in cases of acute illness this is inadmissible. Any form of binder or jacket which can be adjusted with two or three safety pins is preferable to the elaborate bandage, for medical patients at any rate.

Many of our competitors give clear directions about the new poultice being ready before the old one is removed, but hardly anyone speaks of the necesity for covering the poultice with folds of flannel or wadding as well as mackintosh. When the linseed is spread upon linen, and especially when it is made particularly light for application to the abdomen, it is always well to put a good large piece of hot cotton wool or wadding above it, and if this is carefully secured with a binder it keeps warm for some time.

Those candidates who describe each stage of poultice-making are unanimous about shaking the meal into the boiling water, and this is a most satisfactory detail, showing both training and commonsense.

It is characteristic of so-called "Nursing Lectures," delivered by amateurs and even by doctors, that they reverse this order, often saying, "pour the water on to the meal," as is done in the manufacture of some puddings. With fomentations we find that only about half the papers deal really well, some omitting to explain "the wringer," or else forgetting directions for keeping, as well as making, the application hot.

Whilst one person thinks that fomentations should be changed every two hours, another puts five minutes as the due period. The last plan is easier in theory than practice, and we think both patient and nurse would find it impossible of realization.

The importance of preparing the patient for the fresh fomentation is also somewhat inadequately treated, yet it is a point which requires special attention.

If bandages have to be used they should be altogether removed prior to the making of the fresh poultice or fomentation, and of course the patient should be carefully covered over meanwhile.

Most of the papers advise the old application being immediately replaced by the new one, and theoretically this is right. However, many good nurses find it is well to remove the former and rapidly sponge the skin with warm water, dry it perfectly, and cover with a large pad of hot cotton wool before making the new application ready.

This is not only a great comfort to the patient, but it also gives the attendant an opportunity of convincing herself that the skin has received no injury from the repeated hot applications. With a thin patient soreness over bony prominences is apt to occur, and the slightest tenderness with or without redness should be treated with a little oil before the new poultice goes on. It will then be borne with ease by the patient, to whom any soreness, however slight, is a needless aggravation of his sufferings.

In conclusion, we must cordially congratulate ourselves on the proofs we have received that there are a great many excellent poultice-makers amongst our readers, and that the applications made in accordance with these written directions cannot fail to benefit sick people.

We had considerable difficulty in allotting a prize, as so many of the answers this month have some good points, but we have decided to print Sister Matthewson's answer.

Prize Answer.

"In making a linseed-meal poultice, see that the water is thoroughly boiling; next warm the bowl by partially filling with hot water, into which place the spatula that is to be used for mixing. While the bowl is warming, prepare the material on which the poultice is to be spread. In some hospitals old linen or flannel is used, in others tow. If the latter, carefully pull it out and arrange in smooth layers. Having made a sufficiently thick foundation, pour the required quantity of boiling water into the warm bowl, sift the meal through the fingers in case there may be any hard lumps, stirring all the time. When the mixture leaves the sides of the bowl cleanly, it is of a proper consistency, and will not adhere to the patient's skin. Spread evenly and quickly, bearing in mind that heavy poultices are exhausting and uncomfortable. Turn over the edges neatly and apply, rolling the old poultice off as the fresh one is put on, so that chill is avoided. Secure by means of a binder.

"In medical cases poultices should be changed every two or three hours, according to the severity of the case. If stimulants are ordered, it would be well to arrange that the poultices are applied at the hours such stimulants are due, thus counteracting any fatigue that may have been experienced.

"Surgical poultices should be cautiously applied to tender surfaces. If used for a discharging wound, a piece of boracic lint between the wound and poultice acts as an antiseptic and absorbent.

"Fomentations are usually ordered for the relief of pain. They can be made of spongio-piline, lint, or flannel. In the latter cases a piece of jaconet or oil-silk is generally used to retain the heat and moisture. In making a plain fomentation, *i.e.*, without any counter-irritant or anodyne, place the material to be used in the towel or wringer, pour the boiling water over till it is thoroughly saturated; wring quickly till there is no superfluous moisture, shake out, and apply; overlap with the jaconet, cover with cotton-wool, and secure by means of a binder.

"If the pain is severe, renew every half-hour till there is relief, when every hour or more will be sufficiently often.

"Sister Matthewson."

Examination Question for May.

Explain the special nursing required for a case of acute bronchitis (a) in a hospital; and (b) in a private house.

Answers must be sent in by May 3rd. They must be clear and concise, written on one side of the paper only, accompanied by writer's name and address, and directed to "Nursing," Editor of The Hospital.

### Nursing in Australia.

What are the prospects of success for nurses who go out to Australia "on their own account?" This question is frequently brought to our notice, either directly or indirectly.

As regards "success," it depends largely on the ambition of the individual as to its degree. One person asks the question with thought only of "a modest competency," whilst another has vague ideas of the fortunes which once upon a time were to be "picked up" in Australia.

Like all modern hospitals in every quarter of the globe, those in Australia are beginning to train their own probationers, and therefore the demand for nurses "from home" is steadily decreasing.

The colonists are inclined to think that too many women have already gone over there, into the large towns, at any rate, as the doctors have a propensity for employing the newcomers in preference to those trained in Australia. It seems natural enough that the medical men should gladly avail themselves of the assistance of nurses fresh from well-established English training schools, familiar with the last new methods of skilled surgeons and physicians.

Still, we can all see that this bears a little hardly on the nurses already established, and their professional jealousy is constantly aroused by the unequal competition with newly imported Englishwomen.

In the largest towns there are already plenty of private nurses, and the hospital authorities cease to offer passage-money to English-reared women, when trained matrons in Australia are quite qualified to instruct probationers for their own staff. If English nurses, thoroughly trained and certificated, choose to cross the ocean at their own expense and take work "up country" when they arrive, they will have little difficulty in getting it. Although the demand for nurses in towns has been responded to in excess of the need for them, we believe that people living at up-country stations still experience great difficulty in getting skilled women to attend on them in sickness. Nurses who go out to the Colonies must leave a great many comforts behind them; but there is compensation in the free, healthy life and in the cordiality with which their services are welcomed and appreciated. The nurse who dreams of "independence and a moderate income" will probably realise both, but the days of easily-acquired "fortunes" belong to past not to present Colonial experiences.

### LEPERS IN RANGOON.

Mr. Henry C. Moore is appealing for funds for the establishment of a home for lepers in Rangoon. He says the Mission to Lepers in India would gladly start it "but their rapidly extending work and the increasing demands made upon them render it impossible."

### News, Nurses, and Clubs.

The systematic training of nurses has brought quite a new range of subjects before the newspaper readers of to-day. Medical and nursing journals, as well as those which treat of both these closely-allied topics, can be perused at discretion, or they can be avoided by the unprofessional section of the public. But even the last policy will not give security for ignorance of nurses' affairs, because almost all papers, and particularly ladies' journals, dabble in nursing news nowadays.

A paragraph goes its "daily round," fulfilling its "common task" of instructing or misleading the reader unlearned in mysterious nursing terms.

On the whole, the general public perhaps gains by the general smattering of knowledge it unconsciously absorbs, even if it gets a little sick of discourses on "uniforms," "wrongs," and "rights." The halo of sentiment which once upon a time surrounded skilled nurses has been largely dispersed of recent years.

Perhaps the women who take their walks abroad in caricatures of nurses' garments are chiefly to blame for this. The trailing dresses and unkempt hair and draggled veils are certainly symbols of "what to avoid." It is positively a pleasant surprise when we meet a nurse in a nurse-like costume—"a true nurse" in looks and ways. Not the base imitation whose training and claim to the honourable name are alike imaginary.

Not only have newspapers become involved in the subject of nursing, but clubs have been evolved out of it.

Nurses' clubs have much to recommend them when they are quiet and unobtrusive and well fitted for their ostensible object; in fact, when they are types of a good nurse herself. "The Trained Nurses' Club" in Buckingham Street, Strand, fulfils its aim. It is as steadily worked and as satisfactory in every way as when it was first started. The monthly winter lectures are good, clever, and instructive, and not of the "frothy and popular" style distasteful to earnest workers.

There is also a newer club in Charlotte Street, Fitzroy Square, where nurses can live or be outside members, according to their tastes and requirements.

The R.B.N.A. have a nurses' club of their own. Yet another club is in process of formation by Mrs. Stubbs, Beresford House, Beaumont Street, who proposes to start a convenient and comfortable Home for nurses, which will combine many advantages for resident and non-resident members. The letter published by our contemporary *Truth* speaks of there being as yet no public club or recreation rooms open to nurses, but those establishments already in existence, to which we have referred, and, doubtless, others also, provide these facilities, and Mrs. Stubbs has herself been a member of the Trained Nurses' Club which is so conveniently situated in Buckingham Street.

However, as nurses are steadily increasing in numbers, they will need more and more accommodation, and there is doubtless room for another and larger residential club than those already started.

### KEIGHLEY SCIENCE AND FINE ART EXHIBITION.

There is to be a Doll Dressing Exhibition at Keighley in June, and the proceeds will go to furnish the new Children's Ward at the Cottage Hospital. The Duke and Duchess of Westminster and the Bishop of Ripon are amongst the patrons, and the Mayor and Mayoress head the committee. Under such favourable auspices the proposed exhibition will surely be successful, and we hope it will bring a handsome sum of money to the Cottage Hospital funds.

### Rescued.

A weird sound of heavy chains rattling overhead, a crashing, deafening roar from the working of many stamping machines, crushing the stone that the ore may be extracted from it, innumerable rushing tramcars carrying the tin-stone from the mouth of the shaft to the stamping floors. We are on the surface of the oldest, the deepest, the richest, the most wonderful tin mine in the whole world. Hundreds of women, girls, and boys are washing the ore on the tin floors, wheeling it in barrows and arranging it in even, oblong heaps. Tall quaint engine-houses surround us, their powerful engines pumping the water out of the mine night and day. Huge mounds of refuse, sheds, drying-houses, machinery of all kinds and description, from the great steam-engines to the bucket or kibble which brings up the tin-stone and tips it into the train, may be seen on all sides. Such is Dolcoath, where the deepest level is a full half-mile from the surface of the earth, a mine which has yielded up ore worth six millions, which employs sixteen hundred work people, and has been worked with varied fortunes for over a hundred years.

The day of the Cornish miner is divided into three equal portions of eight hours, and no man works underground more than eight hours out of the twenty-four.

In the miners' own language, the twenty-four hours are divided into three "chores"—the first, or "morning chore," beginning at six; the next at two in the afternoon; and the third, or "night chore," at ten.

It is two o'clock in the afternoon; the men are standing about in groups round the mouth of the shaft waiting to go down; there are about a dozen more to come up, and already most of the men who have come up, having finished their morning chore, are washing and exchanging their underground flannel suits for what may be called their above-ground dress, tweed or cloth, more or less well worn and shabby, but rarely ragged. The Cornish miner is sober and thrifty, and he will go home and work in his garden, and the windows of his cottage are bright with well cared-for, luxuriantly-flowering plants. He is blessed with a great love of flowers, and his favourite method of expressing his gratitude to the district nurse is by a present of some cherished plant or the friendly offer of a day or two of work in her tiny garden. There is some delay, but at last six more men appear at the mouth of the shaft; their faces are pallid beneath the grime, and one or two have a look of absolute terror. Those nearest press close round them, while we on the outside of the crowd can only catch the ominous word, "Fire!" The timbering of the shaft is on fire, it is spreading with fearful speed, the strong up current of air is fanning it like a bellows; and seven comrades, neighbours and friends of the men around, are down in the awful depths of Dolcoath, with a raging fire between them and the light of day—between them and their wives and children.

Unless rescued at once, what a death will be theirs; and nothing can be done to extinguish or cope with the fire till they are rescued, dead or alive. Who will volunteer to go down in the cage, brave the fiery furnace, pass through it, and in their turn have it between them and all they hold dearest?

Already a thick column of smoke is pouring up out of the mouth of the shaft; a few more precious minutes and the hope of even an attempted rescue will be gone. Eight young unmarried men come forward; they are willing to go. The peril is too awful for their fellows to give them a cheer.

But a sound goes up from the great crowd, partly a groan and partly a prayer. The manager looks round anxiously; he wants someone to go down with them—someone who is so familiar with the workings of the mine that he will know how to take advantage of every chance.

After a second only of hesitation, a man in the prime of life, with handsome, well-cut features, and clear, resolute eyes, says, "Captain, I will go with them." You would choose him from a regiment of soldiers to lead a forlorn hope. He has a wife and three curly-headed, blue-eyed children at home, he dare not stop to think of them. He only remembers that he has worked in Dolcoath ever since he was a boy, that he knows every inch of the way, and that there are seven comrades to be rescued. He listens to a few words of advice given in a low tone by the manager, then leads the way into the great cage, which, with the nine men in it, begins to descend. Down they go into the very jaws of death, through the stifling smoke, past the burning timber; should one of those beams fall across the shaft they will see the light of day no more.

Up on the surface the immense throng waits in a silence so deep that it strikes the heart with fear, not a whisper is heard. Minutes seem hours, the smoke grows thicker, the smell of the burning timber stronger, the fire is gaining ground rapidly. The thought in each heart is no man can have lived through it. But listen! That is the signal for the cage to be drawn up; the women begin to sob, some words pass between the men, and in a few minutes, long though it seems, the cage slowly comes up out of the shaft; hands that tremble, assist to get them out, eager shouts from the outside of the crowd come through the smoky air, "How many?" And the triumphant answer goes back, "All saved." Rescued and rescuers all safe, some of the former half-suffocated with smoke, one hurt by a falling beam, and one badly burnt, but not a life lost. And when the district nurse pays a visit last thing in the evening to her burnt patient, she finds the handsome leader of the forlorn hope sitting by his bedside chatting softly to him, and very eloquent is the silent handshake that they give each other as he rises to go. This is what happened in Dolcoath two years ago. Has England no Victoria Cross for the men who save life in the face of such fearful odds? F. M. Ll.

### Appointments.

[It is requested that successful candidates will send a copy of their applications and testimonials, with date of election, to The Editor, The Lodge, Porchester Square, W.]

Co. Clare Infirmary.—Miss B. Sigerson has been appointed Matron of Co. Clare Infirmary. Miss Sigerson was trained at the City of Dublin Hospital, Bagot Street, and afterwards worked at Barrington's Hospital, Limerick.

Middlesboro' Fever Hospital.—Miss K. Bell has been appointed Matron of the Middlesboro' Fever Hospital. She was trained at Brownlow Hill, Liverpool, and for the past three years has held the post of Sister and Night Superintendent at Monsall Fever Hospital. We wish her every success, and congratulate the Committee of tho above hospital on securing her services.

Isolation Hospital, Norwich.—Miss Lilla Bayne has been appointed Matron of the Isolation Hospital, Norwich. Miss Bayne was trained at the London Fever Hospital, Islington, and was afterwards Charge Nurse at Monsall Fever Hospital; Night Superintendent at the Fever Hospital, Homerton; and Head Nurse of enteric wards at Bradford. We congratulate the hospital at Norwich on securing the services of a lady who has had such valuable experience.

Edinburgh City Hospital.—Miss Sandford has been appointed Lady Superintendent of Edinburgh City Hospital. Miss Sandford was trained in Tasmania, and also at the Royal Infirmary, Edinburgh, where she worked for three years. Miss Sandford was afterwards Sister at King's College Hospital for four years, and holds the L.O.S. diploma. She has recently given a successful course of lectures on "Home Nursing" for the Technical Education Committee at Springfield, Essex.

### The Book World for Women and Nurses.

[We invite Correspondence, Criticism, Enquiries, and Notes on Books likely to interest Women and Nurses. Address, Editor, The Hospital (Nurses' Book World), 428, Strand, W.C.]

An Old Woman's Outlook. By C. M. Yonge.

These papers, reprinted from the "Monthly Packet," contain reminiscences extending over many years of the beautiful villages near Winchester, which will always be associated with the authors of "The Heir of Redclyffe" and "The Christian Year." It is interesting to have] Miss Yonge's impressions, even in this fragmentary form, of some of the social changes which she has faithfully- reflected in many volumes. The current of opinion has changed indeed since her early books, where the curious may learn the attitude adopted by "nice women" towards such innovations as waltzing, or the little close bonnets which have replaced]the more modest "poke." Miss Yonge has always painfully endeavoured to be fair to the modern]girl, and as a new type of modern girl makes its appearance about once every five years, her novels present a curious series of the small shocks offered to the elder generation by each succeeding variety. In heart she remains faithful to the gentle, self-contained girl, strong in endurance, controlled in feeling, for whom self-restraint was the most important factor in life. She has lived to see this old-fashioned virtue gradually deposed from the place of honour, and deliberately ignored in education, until the baffled doctors (witness a recent article on "Fasting" in The Hospital) discover it to be the true preventive and only cure for half the women's ailments of the day. To return to the "Outlook." At a time when the condition of the village poor is exercising all minds it is good to be reminded that progress, though slow, has been continuous. In Miss Yonge's youth "Union doctors were not. Parishes were supposed to be attended by busy surgeons, but this came to nothing. The lady in each parish had to be doctor, and make the best of the remarkable complaints she heard of. I remember a great white jug where 'Jesuits' bark' was soaked before quinine in powder was cheaply attainable for the ague, which was then common in the parish. But the cure my mother thought the greatest was of a man of whom it was reported that the doctor said his liver was no bigger than a pigeon's egg, and he might take what he pleased, so would she send an 'imposing draught.' My father really knew a good deal about medicine and sent a dose of calomel, and the man recovered. For ague one prescription in the next village was, among others, to have a bandage round the wrists lined with gunpowder and set on fire; or to be led to the top of a mound and violently pushed down. As a remedy for fits, to wear a ring of beaten sixpences given by six young women who had married without changing their surnames, or to wear suspended from the neck 'a hair from the cross on the back of a donkey.' Moreover, a gentleman's butler feeling a lump or rising in his throat, swallowed shot to 'keep down his lights.' Chaney—crushed porcelain—was a favourite remedy." The surgery was equally drastic in those days. "Whole families used to turn out together to reap and bind, and it was considered lucky if the child just promoted to reaping cut herself with the sickle. Even if the top of a finger was cut off it was speedily joined on with a quid of tobacco, according to the remarkable practice of surgery which pre-

### The Hospital Advertiser.

### ROYAL NATIONAL PENSION FUND FOR NURSES.

### THE BOOK WORLD FOR WOMEN AND NURSES—*continued.*

vailed before the days of union doctors. Those were trifles." The book is full of pleasant notes on birds, beasts, and flowers, and abounds in bits of folk-lore. It can be cordially recommended to all lovers of nature.

North Midland School Cookery Book. (Raithly, Lawrence, and Co., London.)

When the Englishman takes up a subject he seldom drops it until he has given it a fair trial. Practical education is now in the crucible, and we await the results. Cookery classes are becoming universal, but the quality of the teaching makes it likely that the results will be very unequal. The poor, and, indeed, the ignorant in all classes need an education to grasp the importance of the theories which are poured helter-skelter into their ears during the course of a dozen or so lectures, accompanied with the minimum, if any, of practical tests. Teaching in cookery, to be of any practical use, must dwell on the minute details of the elementary portion of the subject. Knowledge of the simplest kind must not be taken for granted. Would-be teachers will do well to study the little book, price only sixpence, published for the North Midland School of Cookery. It contains most excellent suggestions for the teaching of the subject, which cannot fail to be of value. The matter of cleaning the kitchen and utensils is not overlooked, and prices and weights are carefully dealt with. The recipes are good and clearly given, though this is not a part of the subject in which a want conspicuously exists, and we ourselves consider the South Kensington system of setting forth recipes to be the most explicit and easily followed of any. The book closes with a short series of recipes for the sick room, which are good if few in number. The little book is worth far more than the humble sixpence asked for it, and reflects credit on the intelligence of the compiler.

The Life Register. (West, Newman, and Co., London.)

Dear to most women are the records which denote the landmarks, so to speak, of life. Not so very long ago almost every young lady kept a diary into whose receptive but uncommunicative bosom she poured her sentiments, sorrows, joys, and imaginations. In our days of rush and hurry, diaries are rather at a discount. Yet there are many who regret that they have not some record of events. It is such a triumph and comfort to place one's finger upon a right date. The diary nowadays seems to reveal itself chiefly in our Courts of Justice, but even such unpleasant notoriety will not extinguish records of events which often prove useful in a more pleasant atmosphere. We have before us a little book called "The Life Register," which, if better known, would prove, we feel sure, very acceptable to mothers. It is especially arranged that the chief and any other events desired in a life from babyhood upwards may be briefly recorded. During the years of childhood suggestions are made as to useful points in health development and characteristics for the mother to note. Indeed, were such systematic observations carefully carried out in the case of children by all mothers, the early indications of disease might be detected and treated, whereas the disease too often only discovered when too late. Following the pages to be devoted to the register of events are some useful hints on the management of health, and is one which would certainly prove useful to many.

